# Cavernous hemangioma originating from the cervical vagus nerve masquerading as a schwannoma: a case report

**DOI:** 10.3389/fsurg.2025.1703168

**Published:** 2026-01-15

**Authors:** Yiyang Lu, Guochen Zhu, Jianxin Hu, Hui Lv, Yan Xiao

**Affiliations:** 1 Department of Otorhinolaryngology-Head and Neck Surgery, Jiangnan University Medical Center (Wuxi NO.2 People’s Hospital), Wuxi, China; 2 Department of Pathology, Jiangnan University Medical Center (Wuxi NO.2 People’s Hospital), Wuxi, China

**Keywords:** case reports, cavernous hemangioma, neck, schwannoma, vagus nerve

## Abstract

Peripheral nerve cavernous hemangioma refers to cavernous vascular malformations occurring on peripheral nerves outside the cranial and spinal nerves. It is a rare non-neoplastic condition. We report a case of a 53-year-old female patient who presented with a painless mass in the right mid-lower neck and a foreign body sensation in the pharynx for five months. Preoperative ultrasonography (US), computed tomography (CT), and magnetic resonance imaging (MRI) revealed a well-delineated lesion with minimal blood flow signals, heterogeneous enhancement on contrast-enhanced CT and T1-weighted imaging, and high signal intensity on T2-weighted imaging. The patient underwent complete surgical excision of the lesion. Intraoperatively, the lesion was found to be located between the epineurium and perineurium of the vagus nerve and was completely enucleated. Postoperative histopathological and immunohistochemical analyses confirmed the diagnosis of cavernous hemangioma. The patient recovered without complications and exhibited no hoarseness or other neurological deficits postoperatively. No signs of lesion recurrence were observed during a 28-month follow-up. This case suggests that when managing tumors of the cervical vagus nerve, vascular lesions should be included in the differential diagnosis, although such instances are relatively rare.

## 1 Introduction

Cavernous hemangioma is a benign vascular lesion composed of abnormally dilated, thin-walled vascular cavities (cavernous spaces) and is often classified as a congenital venous malformation. Histologically, it is characterized by multiple, variably sized, blood-filled cavernous or cystic vascular spaces lined by a single layer of flattened endothelial cells and lacking supporting smooth muscle or elastic fibers, which predispose them to leakage or hemorrhage events (1). Current understanding suggests that its pathogenesis involves the synergistic effects of genetic mutations, endothelial cell dysfunction, and microenvironmental dysregulation (2). Additionally, regarding cellular origin, single-cell transcriptome analysis has revealed that cavernous hemangioma tissues not only contain functionally abnormal endothelial cells (CHECs) but are also enriched with mesenchymal stem cells (MSCs) possessing multidirectional differentiation potential. This finding implies that the abnormal recruitment or differentiation of stem cells in the local microenvironment may contribute to lesion maintenance and progression (3).

Cavernous hemangiomas of peripheral nerves are vascular lesions originating from intraneural vascular components, characterized by abnormally dilated, thin-walled blood vessels that may compress or infiltrate nerve fibers, potentially leading to neurological dysfunction (4). Cavernous hemangiomas occur more frequently in the central nervous system, while their intraneural development in peripheral nerves is extremely rare, with only a limited number of cases reported in the literature (5–8). Clinically, these cases resemble peripheral nerve schwannomas. In the early stages, patients often do not exhibit obvious clinical symptoms. As the lesion gradually enlarges, the symptoms mainly depend on its location and the occurrence of hemorrhage, which may manifest as a palpable mass accompanied by pain, paresthesia, occasional weakness or paralysis, or compressive symptoms in the case of larger lesions. Imaging examinations, particularly MRI, are useful for determining the location and size of the mass. Based on published literature, surgery is considered the most successful and effective treatment (9).

To date, there have been no reports of cavernous hemangioma specifically originating from the cervical vagus nerve. Herein, we report a case of cavernous hemangioma originating from the cervical vagus nerve masquerading as a schwannoma. Although this condition is exceedingly rare, it should be considered in the differential diagnosis of vagus nerve tumors, and efforts should be made to preserve the integrity of the vagus nerve during surgery.

Informed consent was obtained from the patient, and approval was granted by the local ethics committee.

## 2 Case presentation

**Figure 1:**
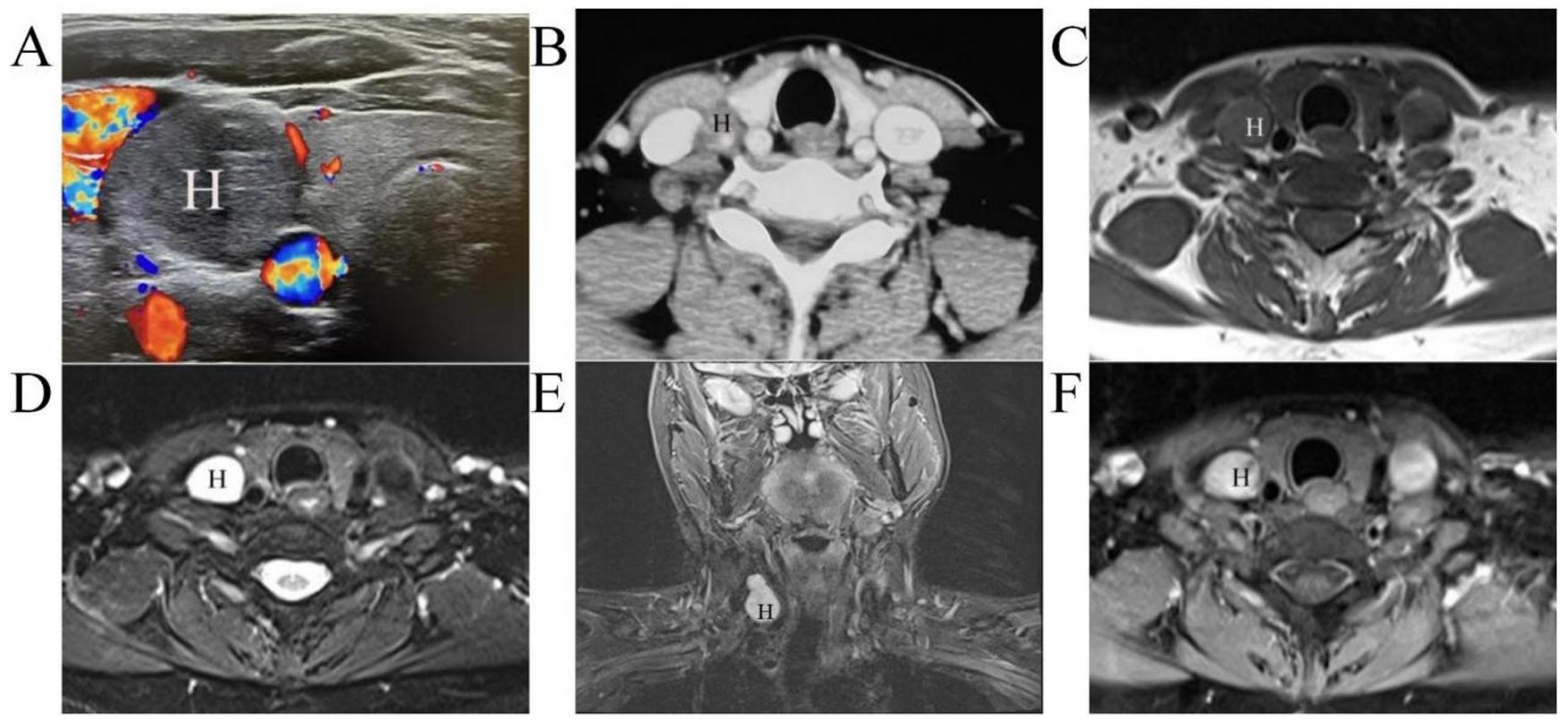
Preoperative imaging of the neck lesion. Ultrasonography of axial section **(A)** Enhanced computed tomography of axial section **(B)** T1-weighted imaging of axial section **(C)**; T2-weighted imaging with DIXON fat suppression of axial section **(D)**, and TIRM fat suppression of coronal section **(E)**; enhanced T1-weighted imaging with DIXON fat suppression of axial section **(F)** H, cavernous hemangioma.

A 53-year-old female patient was admitted to the hospital due to a “neck mass with a foreign body sensation in the pharynx for five months.” Five months ago, the patient incidentally discovered a neck mass in the right mid-to-lower cervical region. She began to experience a significant foreign body sensation in the pharynx, without associated symptoms such as cough, neck pressure, dyspnea, dysphagia, hoarseness, or facial numbness. The patient had not received any surgical, radiological, or medical interventions for the neck mass before this presentation. The worsening pharyngeal discomfort prompted her visit to our hospital. Her past medical history included a cesarean section at age 23. She had no history of chronic diseases, no long-term medication use, and no family history of hereditary diseases. Physical examination revealed a palpable mass measuring approximately 35 mm × 15 mm in the right mid-to-lower neck, which did not move with swallowing. The mass was soft, well-circumscribed, non-tender, and there were no palpable enlarged lymph nodes in the neck. The thyroid gland was non-palpable, and the trachea was midline. Oral examination and cranial nerve assessment (including vagus nerve function) were normal, and the systemic review was otherwise unremarkable. Laboratory tests, including thyroid function tests, complete blood count, and routine biochemistry panels were all within normal limits. Ancillary examinations: Cervical ultrasonography (US) demonstrated a well-defined mass measuring approximately 35 mm × 20 mm × 15 mm in the right mid-to-lower neck region, and located between the internal jugular vein and the common carotid artery. The internal jugular vein was compressed and flattened, and the common carotid artery was displaced medially. US demonstrated minimal vascularity within the mass (Figure 1A). Non-enhanced CT showed isodensity, with mild heterogeneous enhancement observed on contrast-enhanced CT (Figure 1B). On MRI, the mass exhibited isointensity on T1-weighted images (T1WI, Figure 1C) and heterogeneous hyperintensity on T2-weighted images (T2WI) with DIXON or TIRM fat saturation (Figures 1D,E). Post-contrast T1WI with DIXON fat suppression revealed heterogeneous enhancement, comparable to the signal intensity of the contralateral internal jugular vein (Figure 1F). Thyroid ultrasound revealed no significant abnormalities. The preoperative radiological diagnosis was schwannoma, likely originating from the cervical vagus nerve.

**Figure 2:**
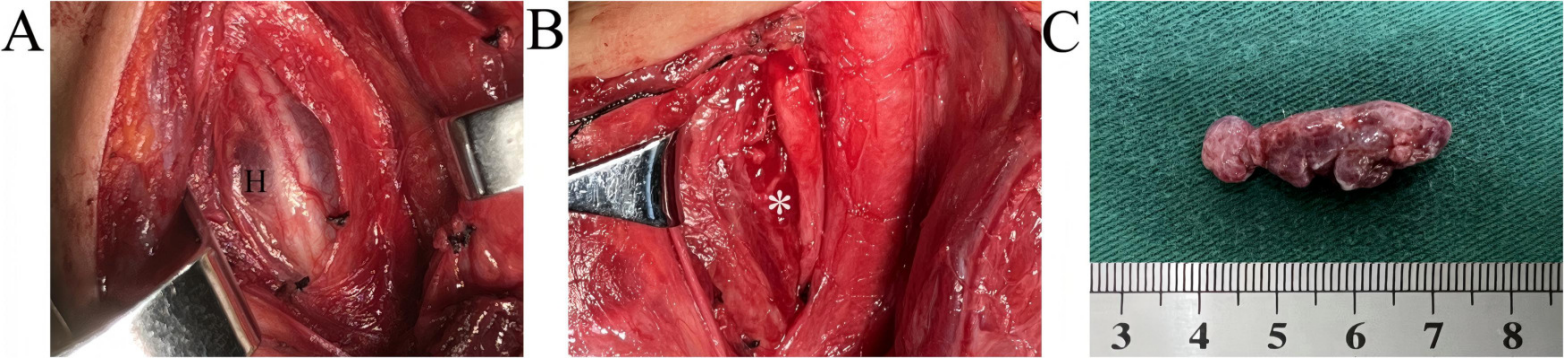
Intraoperative pictures of dissecting the neck lesion. **(A)** Exposure of the mass; **(B)** After total enucleation of the mass; **(C)** General view of the mass. H: cavernous hemangioma; *site of the mass.

Following a multidisciplinary discussion, surgical enucleation of the tumor with maximal nerve preservation was planned. Under microscopic visualization, a standard pre-sternocleidomastoid incision was performed. The subcutaneous tissue, platysma, omohyoid muscle, sternohyoid muscle, and sternothyroid muscle were sequentially exposed, and the carotid sheath was dissected using a combination of blunt and sharp techniques. Intraoperatively, a dark red mass was identified between the internal jugular vein and common carotid artery. It was adherent to the epineurium on the dorsal aspect of the right mid-to-lower cervical vagus nerve (Figure 2A), displacing the common carotid artery medially and dorsally relative to the thyroid gland. A longitudinal incision was made in the posterolateral epineurium, resulting in minimal bleeding of dark red blood. The mass was completely excised from caudal to cephalad directions while preserving the perineurium (Figure 2B). Intraoperative findings confirmed that the mass was located within the epineurium, but outside the perineurium of the vagus nerve. Macroscopically, the resected specimen was soft and gourd-shaped (Figure 2C), measuring approximately 30 mm in length and 12 mm in maximum transverse diameter.

The patient's surgical specimen was processed into 4 µm thick, paraffin-embedded (Shanghai Yiyang Medical Equipment Co., Ltd., Shanghai, China) sections for histopathological and immunohistochemical staining. All primary antibodies (ready-to-use) were obtained from Maxim Biotech Inc. (Fujian, China). In brief, sections were dewaxed in xylene (Xilong Chemical Co., Ltd., Shantou, China), dehydrated through a graded ethanol series, and rinsed in distilled water for 1 min. Following rehydration, antigen retrieval was performed as required for each primary antibody. The slides were then incubated with one drop (approximately 50 µL) of primary antibody at 4 °C overnight, followed by incubation with a secondary antibody (approximately 50 µL) from the Elivision™ Plus Poly-HRP (Mouse/Rabbit) IHC Kit at room temperature for 30 min. Color development was performed using Ultra Diaminobenzidine (Maxim Biotech Inc., Fujian, China). After each of the above steps, the slides were washed three times (3 min each) with phosphate-buffered saline (Maxim Biotech Inc., Fujian, China). Finally, the sections were counterstained with hematoxylin and eosin (Maxim Biotech Inc., Fujian, China), dehydrated through an ethanol series, cleared in xylene, and coverslipped (Maxim Biotech Inc., Fujian, China) for observation under a light microscope (BX-51, Olympus, Japan).

**Figure 3:**
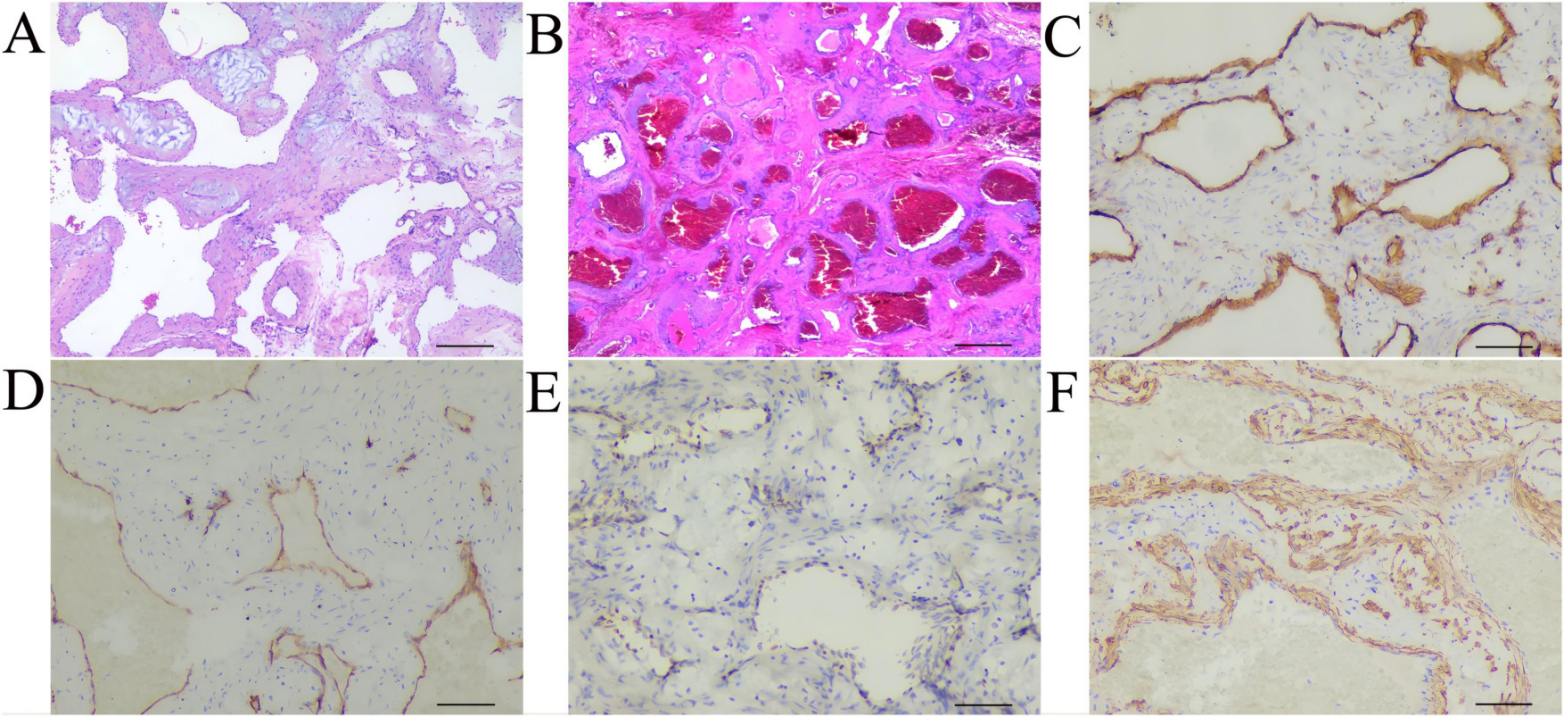
Pathological detection of the mass (scale, 50 μm). Hematoxylin and eosin staining showed that the mass consisted of vascular spaces and the cavities were full of blood and thrombi **(A,B)**. Immunohistochemical analysis showed positive expression of CD31 **(C)**, CD34 **(D)** and friend leukaemia integration-1 **(E)** in the monolayer of flattened endothelial cells, and smooth muscle actin in vascular walls **(F)** Pathological detection of the mass confirmed a cavernous hemangioma.

Hematoxylin and eosin staining revealed numerous dilated, thin-walled vascular spaces (Figures 3A,B). Immunohistochemistry showed positive staining for CD31, CD34, and Friend Leukemia Integration-1 in the endothelial cells, and smooth muscle actin in the vascular walls (Figures 3C–F). The vascular spaces contained blood and thrombi; endothelial cells showed mild proliferative activity without atypia or pathological mitoses. Some vascular channels anastomosed to form papillary-like structures with collagenous or myxoid stromal cores. The lesion was negative for S-100 protein and D2-40 (not shown), and the Ki-67 proliferation index was less than 1%. No nerve fibers were identified within the specimen. The final pathological diagnosis was extrafascicular cavernous hemangioma of the cervical vagus nerve.

**Figure 4:**
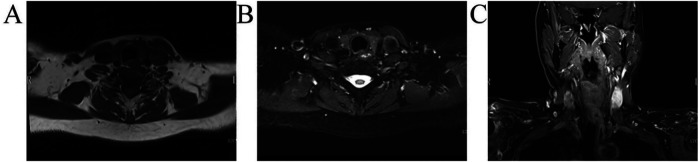
Postoperative imaging of the neck lesion at 28 months. T1-weighted imaging of axial section **(A)**; T2-weighted imaging with DIXON fat suppression of axial section **(B)**; enhanced T1-weighted imaging with DIXON fat suppression of coronal section **(C)**.

After surgery, the patient recovered uneventfully without hoarseness or other neurological deficit. The patient reported, “Preoperative anxiety and fear caused my insomnia. After the surgery, the foreign body sensation in my throat has significantly disappeared, and I have no other discomfort apart from incision pain.” Follow-up examinations by MRI over 28 months showed no evidence of tumor residue or recurrence (Figure 4).

## 3 Discussion

Based on the extent of neural involvement, peripheral nerve cavernous hemangiomas can be classified into two types (4, 10). Type I lesions are extrafascicular and can typically be completely resected without nerve damage, leading to prompt or gradual symptom relief. In contrast, Type II lesions are intrafascicular and encase the nerve fascicles, making complete resection challenging and potentially resulting in neurological deficits. In such cases, nerve grafting may be required (11, 12). To reduce the risk of severe complications, subtotal resection may be considered, although this approach carries a higher risk of disease progression or recurrence (13). In the present case, intraoperative findings confirmed a Type I lesion. The mass was completely removed without nerve injury, and the patient's pharyngeal foreign body sensation gradually resolved.

The initial symptom of peripheral nerve cavernous hemangioma is typically a painless, slowly growing mass, with growth often spanning years. As the cavernous hemangioma enlarges, it can gradually compress sensory nerves, leading to sensory abnormalities (such as numbness, tingling, etc.). When motor nerve fibers are compressed, it may result in weakness and atrophy of the innervated muscles (14). Therefore, asymptomatic cases may be managed with observation. However, when symptoms arise, a reasonable surgical plan should be formulated based on the classification of the peripheral nerve cavernous hemangioma. Surgery must be performed with extreme caution, as the lesion often adheres tightly to the nerve, making it difficult to achieve both complete resection and functional preservation. In some cases, intracapsular resection or subtotal resection may be necessary to alleviate symptoms.

Preoperative diagnosis of cavernous hemangioma of the cervical vagus nerve poses a significant challenge (4, 15–19). Firstly, the clinical history and symptoms are often non-specific. In the present case, the patient presented solely with a neck mass accompanied by a pharyngeal foreign body sensation, without concomitant symptoms such as cough, dyspnea, dysphagia, or hoarseness. Secondly, while imaging examinations can determine the tumor's location and potential neural origin, and in this case helped rule out thyroid-related pathologies, distinguishing cavernous hemangioma from other vagus nerve tumors (e.g., schwannoma, paraganglioma) and lymphatic malformations remains difficult. Dynamic contrast-enhanced MRI is crucial for differentiation. Paragangliomas, due to their rich vascularity, appear isointense or hypointense on T1WI and markedly hyperintense on T2WI, exhibiting the “salt-and-pepper” appearance, where the “pepper” represents the abundant flow voids within the tumor and the “salt” represents the hyperintense tumor parenchyma, with significant homogeneous enhancement on contrast-enhanced scans (20). Lymphatic malformations, on the other hand, present as well-defined or poorly defined cystic lesions, hypointense to isointense on T1WI, markedly hyperintense on T2WI, with possible mild enhancement of the cyst walls and no enhancement of the contents on contrast-enhanced scans, consistent with their cystic fluid nature (21). The imaging features in this case were largely consistent with those of vagus nerve schwannomas, including well-defined margins, minimal vascularity, heterogeneous enhancement on contrast-enhanced CT or T1WI, and high signal intensity on T2WI. Therefore, although dynamic contrast-enhanced MRI is a valuable tool, definitive differentiation from schwannomas can be challenging (22).

In a previously reported case, Tzortzis et al. described a 41-year-old male patient presenting with a non-tender mass in the right neck. Preoperative imaging suggested a cervical vagus nerve schwannoma, and the tumor was successfully and completely dissected from the vagus nerve during surgery, with the diagnosis later confirmed by histopathology (23). In the present case, preoperative imaging findings closely resembled those of the aforementioned report, leading to a similar preoperative suspicion of a vagus nerve-derived schwannoma. Intraoperatively, clear anatomical localization confirmed the lesion's origin from the vagus nerve, excluding involvement of the accessory nerve, hypoglossal nerve, or other adjacent neural structures. Since schwannomas originate from Schwann cells, immunohistochemistry plays a pivotal role in differentiating them from other spindle cell tumors. S-100 protein, a marker of neural crest-derived cells, is strongly and consistently expressed in the vast majority of schwannomas, as supported by multiple studies (24), making it one of the most sensitive and specific immunohistochemical markers for diagnosis. However, postoperative histopathological examination in our patient revealed morphological features consistent with cavernous hemangioma. Hematoxylin and eosin staining showed numerous dilated, thin-walled vascular channels lined by a single layer of endothelial cells. Immunohistochemistry demonstrated positive expression of CD31 and CD34 in endothelial cells, along with diffuse positivity for smooth muscle actin in pericytes surrounding the vascular walls, indicating a mature vascular architecture. In contrast, the lesion was negative for both S-100 protein and D2-40, effectively ruling out schwannoma and lymphangioma, respectively. Notably, the negativity for D2-40 helped exclude the possibility of a lymphatic origin within the vascular system (25). In conclusion, despite clinical and imaging features highly suggestive of a vagus nerve schwannoma, definitive diagnosis in this case ultimately relied on comprehensive postoperative histopathological assessment (26).

In summary, the differential diagnosis of neurogenic or neurogenic-like masses in the head and neck region requires a comprehensive evaluation integrating imaging, intraoperative findings, and histological characteristics, with particular emphasis on the auxiliary value of immunohistochemical markers to avoid misdiagnosis and inappropriate management. Furthermore, cavernous hemangioma should be considered in the differential diagnosis of vagus nerve tumors, despite the rarity of such lesions, particularly in cases presenting as painless neck masses. Comprehensive preoperative imaging assessment with US, CT, and MRI is essential. During surgery, careful confirmation of the relationship between the mass and the nerve sheath is critical. Postoperative diagnosis relies on histopathological examination of tissue sections. Regarding treatment, as the hemangioma can cause neurological symptoms through compression, microsurgical complete resection with preservation of nerve integrity is a recommended and feasible approach for extrafascicular (Type I) hemangiomas. This strategy aims to achieve a cure while avoiding permanent neurological impairment.

## Data Availability

The original contributions presented in the study are included in the article/Supplementary Material, further inquiries can be directed to the corresponding author.
