# Supplementary material for: Cavernous hemangioma originating from the cervical vagus nerve masquerading as a schwannoma: a case report
Source: Front Surg. 2026 Jan 15;12:1703168. doi: 10.3389/fsurg.2025.1703168 (PMC12852428; doi:10.3389/fsurg.2025.1703168)
Supplement: Supplementary file 1 [file Supplementaryfile1.pdf]

CARE Checklist of information to include when writing a case report

CARE case report guidelines

(cc) BY-NC-ND

Topic	Item	Checklist item description	Reported on Line
Title	1	The diagnosis or intervention of primary focus followed by the words "case report"	Title
Key Words	2	2 to 5 key words that identify diagnoses or interventions in this case report, including "case report"	Key Words
Abstract (no references)	3a	Introduction: What is unique about this case and what does it add to the scientific literature?	Abstract
	3b	Main symptoms and/or important clinical findings	Abstract
	3c	The main diagnoses, therapeutic interventions, and outcomes	Abstract
	3d	Conclusion—What is the main "take-away" lesson(s) from this case?	Abstract
Introduction	4	One or two paragraphs summarizing why this case is unique (**may include references**)	Introduction
Patient Information	5a	De-identified patient specific information.	Case Presentation
	5b	Primary concerns and symptoms of the patient.	Case Presentation
	5c	Medical, family, and psycho-social history including relevant genetic information	Case Presentation
	5d	Relevant past interventions with outcomes	Case Presentation
Clinical Findings	6	Describe significant physical examination (PE) and important clinical findings.	Case Presentation
Timeline	7	Historical and current information from this episode of care organized as a timeline	Abstract
Diagnostic Assessment	8a	Diagnostic testing (such as PE, laboratory testing, imaging, surveys).	Case Presentation
	8b	Diagnostic challenges (such as access to testing, financial, or cultural)	Case Presentation
	8c	Diagnosis (including other diagnoses considered)	Case Presentation
	8d	Prognosis (such as staging in oncology) where applicable	Case Presentation
Therapeutic Intervention	9a	Types of therapeutic intervention (such as pharmacologic, surgical, preventive, self-care)	Case Presentation
	9b	Administration of therapeutic intervention (such as dosage, strength, duration)	Case Presentation
	9c	Changes in therapeutic intervention (with rationale)	Case Presentation
Follow-up and Outcomes	10a	Clinician and patient-assessed outcomes (if available)	Case Presentation
	10b	Important follow-up diagnostic and other test results	Case Presentation
	10c	Intervention adherence and tolerability (How was this assessed?)	Case Presentation
	10d	Adverse and unanticipated events	Not Applicable
Discussion	11a	A scientific discussion of the strengths AND limitations associated with this case report	Discussion
	11b	Discussion of the relevant medical literature **with references**.	Discussion
	11c	The scientific rationale for any conclusions (including assessment of possible causes)	Discussion
	11d	The primary "take-away" lessons of this case report (without references) in a one paragraph conclusion	Discussion
Patient Perspective	12	The patient should share their perspective in one to two paragraphs on the treatment(s) they received	Case Presentation
Informed Consent	13	Did the patient give informed consent? Please provide if requested	Yes ☑ No ☐
